# Potassium Concentration on Admission Is an Independent Risk Factor for Target Lesion Revascularization in Acute Myocardial Infarction

**DOI:** 10.1155/2014/946803

**Published:** 2014-01-12

**Authors:** Tsuyoshi Honda, Kazuteru Fujimoto, Yuji Miyao, Hidenobu Koga, Masanobu Ishii

**Affiliations:** Department of Cardiology, Cardiovascular Center, National Hospital Organization Kumamoto Medical Center, 1-5 Ninomaru, Chuo-ku, Kumamoto 860-0008, Japan

## Abstract

*Background*. Acute myocardial infarction (AMI) is accompanied by excessive production of catecholamines, which is characterized by a hypokalemic dip. A polymorphism of the adrenergic receptor has also been reported to be associated with target lesion revascularization (TLR) after coronary intervention. *Subjects and Methods*. We enrolled 276 consecutive patients with AMI within 24 hours of symptom onset, who underwent emergency coronary intervention using bare metal stents and had examinations over a 5–10-month follow-up period. The patients were divided into tertiles based on their serum potassium level on admission (low K, <3.9; mid K, ≥3.9, <4.3; and high K, ≥4.3). *Results*. Sixty-four TLRs were observed in the study. Increased potassium concentration was associated significantly with TLR. Patients in the high K group were about two and a half times more likely to have a TLR after AMI compared to those in the low K group. Multiple logistic analysis showed that potassium level on admission was an independent risk factor for TLR (odds ratio 1.69; confidence interval 1.04 to 2.74; *P* = 0.036). *Conclusions*. These findings indicated that increased potassium levels on admission might predict TLRs in AMI patients treated with bare metal stents.

## 1. Introduction

Acute myocardial infarction (AMI) is a leading cause of death in adults, with AMI survivors having an increased risk of developing heart failure. It is important to evaluate patient risk such as cardiac remodeling and future adverse events in the early stage after an AMI.

Several pathological conditions are associated with sometimes marked increase in the plasma concentration of catecholamines. This increase is accompanied by hypokalemia [1]. The first well-documented example of this association was in a study of 14 patients with myocardial infarction (MI) [2]. A later study of 1074 patients with acute myocardial infarction (AMI) showed that hypokalemia correlated with ventricular fibrillation [3]. More recently, a study on 517 patients with MI showed that, at admission, only 8% had a low plasma K^+^, with these patients having a significantly higher frequency of ventricular fibrillation [4]. A prospective study on 2428 patients showed that acute coronary syndrome in patients treated with beta blockers was not associated with an early hypokalemic dip seen at the onset of chest pain. This indicated that the hypokalemia was a response to adrenergic activation of the Na^+^, K^+^ pump. Interestingly, in patients with diabetes, the early hypokalemic dip was also shown to be absent, possibly reflecting sympathetic nerve dysfunction, a common complication of diabetes [5]. Attenuation is seen most clearly in unstable angina and is less pronounced in AMI when the early dip in potassium and its later recovery are preserved, albeit with a loss of significance [5]. This may reflect the fact that adrenergic responses to acute coronary syndromes are related to the extent of myocardial injury and are therefore greater in this condition [6]. However, the early dip in serum potassium concentrations has been shown to be attenuated when adrenergic stimulation of sodium-potassium ATPase is blocked, thereby reducing potassium flux across the cell membrane. A previous study also demonstrated that *β*_2_-adrenoceptors mediate the stimulating effect of adrenaline on active electrogenic Na-K transport [7].

Previous studies have shown that a genetic inflammatory factor, the *β*_2_-adrenergic receptor gene (ADRB2), predicts restenosis after percutaneous coronary intervention [8]. The ADRB2 gene has a role in the inflammatory response, with adrenergic receptors on human platelets stimulating ADRB2 to activate platelet nitric oxide synthase (NOS) [9]. NOS catalyzes the formation of NO, which has an inhibitory role on leukocyte adhesion, platelet adhesion and aggregation, smooth muscle cell proliferation, and matrix proteins, and it also has an effect on promoting endothelial survival and proliferation [10].

This study examined the association between plasma potassium concentration on admission and target lesion revascularization in patients with AMI.

## 2. Methods

### 2.1. Study Population

We retrospectively studied 306 consecutive patients who underwent percutaneous coronary intervention (PCI) at Kumamoto Medical Center within 24 hours after the onset of AMI between January 2008 and October 2012 and had a follow-up examination 5 to 10 months later (Table 1). The diagnosis of AMI was made using the universal definition of myocardial infarction [11]. As differences in PCI (thrombolysis, aspiration, plain old balloon angioplasty (POBA), bare metal stents (BMSs), and drug-eluting stent (DES)) may influence TLR in patients with AMI, 30 patients were excluded from the study (1 for thrombolysis, 1 for aspiration, and 28 for DES) [12, 13]. The main exclusion criteria were the presence of malignant disease, severe renal failure, known hepatic disease, and the lack of a follow-up examination. The primary endpoint was TLR after AMI.

The patients were divided into tertiles according to potassium level on admission (low K, <3.9; mid K, ≥3.9, <4.3; and high K, ≥4.3).

Systolic blood pressure (SBP), heart rate (HR), plasma glucose (PG), white blood cell (WBC) count, creatinine, hemoglobin, and potassium were measured at the time of arrival at the Emergency Unit of Kumamoto Medical Center. Creatine kinase (CK) was measured every 4 to 6 hours after admission, and peak CK was calculated in all the patients. Blood samples for measurement of PG, WBC count, creatinine, hemoglobin, and potassium were obtained at the time of hospital admission. Total cholesterol (T-C), high-density lipoprotein cholesterol (HDL-C), low-density lipoprotein cholesterol (LDL-C), triglyceride (TG), and hemoglobin A1c (HbA1c) were measured in the fasting state on the day after admission.

### 2.2. Analysis of Coronary Risk Factors for Coronary Artery Disease

The traditional risk factors for coronary artery disease used in the statistical analyses were hypertension, lipid profiles (T-C, HDL-C, LDL-C, and TG), current cigarette smoking habits, body mass index (BMI), and diabetes mellitus. Current cigarette smoking was defined as ≥10 cigarettes/day for 10 years including cessation of smoking within 1 year, hypertension as blood pressure ≥140/90 mm Hg and/or the use of antihypertensive medication, and diabetes mellitus as a fasting blood glucose ≥126 mg/dL and/or HbA1c ≥6.5% or the use of antidiabetic medication.

### 2.3. Coronary Angiography and Coronary Intervention

Emergency coronary intervention using BMSs was performed in all the patients. The AMI sites were divided into two groups (anterior AMI and nonanterior AMI). A diseased coronary artery was defined as stenosis ≥75%, with the patients being divided into two groups (single-vessel disease and multivessel disease). Reperfusion therapy was used at the discretion of the attending physician.

### 2.4. Target Lesion Revascularization

A clinically driven TLR procedure was defined as one performed because of recurrent angina and/or documented ischemia on noninvasive tests, with >70% diameter stenosis assessed by quantitative coronary angiography (QCA) in the absence of symptoms [14].

### 2.5. Statistical Analysis

The subjects were divided into tertiles based on their serum potassium level on admission. Differences in frequencies were analyzed using the chi-square method. A comparison of continuous variables in the three groups was performed using a one-way analysis of variance (ANOVA), followed by Scheffé's procedure to compare individual groups. Continuous variables were compared in the two groups using a two-tailed paired *t*-test. Odds ratios (OR) and 95% confidence intervals (CI) for target lesion revascularization (TLR) were calculated using logistic regression analysis. Differences with a *P* value <0.05 were considered statistically significant in all the analyses. Data were expressed as the mean ± SD.

## 3. Results

### 3.1. Clinical Characteristics of Patients at Baseline

The patients in the high K group were significantly older than those in the low K group. Although the value for LDL-C was lower in the high K group than in the other groups, there was no difference in the other coronary risk factors and previous medical treatment between the three groups (Table 1).

### 3.2. Clinical and Angiographic Characteristics of AMI

The time between chest pain and admission was longer in the high K group than in the low K group. The incidence of Killip ≥II was increased in the high K group. PG was increased in the high K group compared with the mid K group. WBC count and peak CK were higher in the low K group than in the mid K group. Stent diameter implanted in the culprit lesion was larger in the low K group than in the two other groups (Table 2).

### 3.3. Clinical Characteristics of Patients at Followup

Sixty-four TLRs (23.2%) were observed in the patient group in the present study. The incidence of TLR was increased in the high K group. There were no significant differences in lipid profile and active medical treatment at followup between the three groups (Table 3).

### 3.4. Logistic Analysis for the Risk of Target Lesion Revascularization

Multiple logistic analysis using the variables listed in Tables 1 and 2 showed that admission potassium level (odds ratio, 1.69; *P* = 0.0360; confidence interval, 1.04 to 2.74) was the strongest independent risk factor for predicting TLR (Table 4).

### 3.5. Relationship between Admission Potassium Level and Target Lesion Revascularization

A higher potassium concentration was observed in patients with TLR. Patients in the high K group were two and a half times more likely to have a TLR compared with the low K group (Table 5).

## 4. Discussion

We report for the first time that increased potassium concentration on admission is associated significantly with TLR in patients with AMI.

In high K group, the value of creatinine and the incidence of Killip >I were increased. Renal elimination is the main excretion route of potassium, with approximately 90% being excreted by the kidney. Impaired renal function therefore predisposes to the development of hyperkalemia [15]. In addition, sympathetic nervous system hyperactivity is observed in patients with renal injury, chronic kidney disease, and end-stage renal disease [16]. Sympathetic nerve system activation has been shown to have a proinflammatory and profibrotic effect on the heart and vasculature and contributes to hyperinsulinemia and insulin resistance [16, 17]. These effects might be associated with TLR after AMI. As Killip class is an index of heart failure and cardiogenic shock after AMI, a high Killip class on admission may be associated with a reduction in cardiac output and subsequent low renal blood flow, which may, in turn, reduce renal function and increase serum potassium level [15, 18].

In addition, there was a tendency that the incidence of DM increased in high K group. A previous study reported that ACS patients with diabetes had significantly higher serum potassium concentrations and did not exhibit the early dip seen in nondiabetes. This may reflect sympathetic nerve dysfunction, a common complication of diabetes [5]. Insulin resistance may also have a role in preventing the early dip in serum potassium in diabetes by attenuating intracellular ionic flux in the early stages after onset of symptoms. However, the experimental findings of Brown and colleagues indicate that insulin does not contribute significantly to adrenergically induced changes in serum potassium [19]. Acute coronary syndrome provides a useful clinical model of adrenergic stress as it reflects many of the typical presenting features such as diaphoresis and tachycardia. Hypokalemia has also been attributed to adrenergic stress, and the finding of lower serum potassium concentrations in patients presenting very early after the onset of chest pain is well recognized. So, we excluded AMI patients over 24 hours after onset in the present study.

On the other hand, there was a long delay in presentation to hospital in the high K group compared with the low K group. Past studies reported that aging and DM might be associated with atypical symptoms in AMI and delay in presentation to hospital [20–23]. Older age and the incidence of DM in the high K group might affect the prolonged time from chest pain to admission.

Currently, available BMSs are considerably more effective than older types of BMSs [24]. There is evidence that deployment of a BMS in AMI patients with infarct-related arteries ≥3.5 mm is associated with low rates of TLR [25]. Several studies have shown that diabetes, renal insufficiency, and minimum stent area are risk factors for restenosis after a primary PCI in patients with an AMI [26–29].Hyperkalemia in AMI might be a marker of increased TLR risk in patients with more extensive and diffuse atherosclerotic vascular disease.

Although focus has been paid to potassium concentrations associated with lethal arrhythmias and mortality after an AMI, the present study demonstrated that increased potassium concentrations on admission may be an independent risk factor for TLR after these events [30–32]. A previous study showed that the polymorphism in the ADRB2 gene after adjustment for other variables was a risk factor for restenosis, with the 16A/G polymorphism resulting in an amino acid change from glycine to arginine at position 16 (Arg16Gly) [6]. Patients with homozygosity for the 16Gly variant may have different functional responses to adrenergic stimulation, thereby possibly modulating cardiovascular and metabolic phenotypes. It has been reported that the 16Gly variant of ADRB2 is associated with faster agonist-induced downregulation of the receptor compared with the 16Arg variant [33]. The higher risk of restenosis may be related to less vasodilatation as a result of downregulation of the receptor containing 16Gly compared with the receptor containing 16Arg. Moreover, downregulation of the ADRB2 could result in impaired inhibition of platelet aggregation [8]. Previous studies have reported that the ADRB2 polymorphism may be associated with diabetes, insulin resistance, hypertension, and chronic kidney disease and that subjects with the polymorphism had a high risk for coronary heart disease [34, 35].

It is possible that the ADRB2 polymorphism might prevent the early dip in serum potassium as a result of attenuating intracellular ionic flux early after AMI onset by attenuation of adrenergic stimulation of sodium-potassium ATPase, because a previous study showed that *β*_2_-adrenoceptors mediated the stimulatory effect of adrenaline on active electrogenic Na-K transport [7]. Although we did not investigate the ADRB2 polymorphism in this study, it is possible that hyperkalemia on admission (absence of early potassium dip) in AMI patients may be related to TLR after AMI via absence of the ADRB2 pathway.

## 5. Conclusions

In conclusion, the potassium level on admission for an AMI is an independent risk factor for TLR in AMI patients treated with BMSs.

## Figures and Tables

**Table 1:** Clinical characteristics of the patients at baseline.

	Low K (<3.9)	Mid K (≥3.9, <4.3)	High K (≥4.3)	*P* value
	n = 92	n = 92	n = 92	
Age (yrs)	66 ± 12	67 ± 13	70 ± 12*	
Male (%)	65 (70.7)	68 (73.9)	63 (68.5)	0.716
BMI (%)	23.8 ± 2.8	23.1 ± 2.9	23.6 ± 3.5	
Current smoker (%)	39 (42.4)	34 (37.0)	35 (38.0)	0.727
Hypertension (%)	63 (68.5)	48 (52.2)	56 (60.9)	0.0771
Diabetes mellitus (%)	24 (26.1)	29 (31.5)	38 (41.3)	0.0841
T-C (mg/dL)	198 ± 38	195 ± 39	184 ± 39*	
HDL-C (mg/dL)	44 ± 11	45 ± 12	45 ± 12	
LDL-C (mg/dL)	130 ± 34	126 ± 32	116 ± 33^∗†^	
TG (mg/dL)	129 ± 84	124 ± 76	126 ± 93	
HbA1c (%)	6.0 ± 1.7	5.9 ± 1.3	6.3 ± 1.5	
Previous medical treatment				
ACE-I/ARB (%)	17 (18.5)	21 (22.8)	24 (26.1)	0.581
Beta-blocker (%)	1 (1.09)	2 (2.17)	6 (6.52)	0.105
Diuretics (%)	4 (4.35)	3 (3.26)	7 (7.61)	0.411
Statin (%)	13 (14.1)	14 (15.2)	18 (19.6)	0.573

BMI: body mass index; T-C: total cholesterol; HDL-C: high-density lipoprotein cholesterol; LDL-C: low-density lipoprotein cholesterol; TG: triglyceride; HbA1c: hemoglobin A1c; ACE-I: angiotensin converting enzyme inhibitor; ARB: angiotensin receptor blocker. *P < 0.05 versus low K; ^†^ *P* < 0.05 versus mid K.

**Table 2:** Clinical and angiographic characteristics of acute myocardial infarction.

	Low K (<3.9)	Mid K (≥3.9, <4.3)	High K (≥4.3)	*P* value
	n = 92	n = 92	n = 92	
Time from chest pain to door (hr)	3.9 ± 5.4	5.2 ± 6.4	6.8 ± 6.9*	
SBP on admission (mmHg)	133 ± 30	137 ± 31	131 ± 35	
HR on admission (mmHg)	74 ± 20	74 ± 19	79 ± 27	
Previous MI	4 (4.35)	7 (7.61)	9 (9.78)	0.359
Preinfarction angina	36 (39.1)	36 (39.1)	25 (27.2)	0.146
STEMI	73 (79.3)	70 (76.0)	61 (66.3)	0.111
Anterior MI	50 (54.3)	42 (45.7)	41 (44.6)	0.347
Killip > I	17 (18.5)	13 (14.1)	27 (29.3)	0.0318
Coronary multivessel involvement (%)	35 (38.0)	33 (35.9)	36 (39.1)	0.898
PG on admission (mg/dL)	184 ± 84	169 ± 65	202 ± 114^†^	
WBC count on admission (/mm^3^)	10685 ± 3383	9468 ± 3412*	9998 ± 3477	
Creatinine on admission (mg/dL)	0.79 ± 0.59	0.81 ± 0.33	0.99 ± 0.90*	
Hemoglobin on admission (g/dL)	14.0 ± 1.6	13.8 ± 2.1	13.6 ± 2.2	
Potassium on admission (mEq/L)	3.6 ± 0.2	4.0 ± 0.1*	4.7 ± 0.6^∗†^	
Stent diameter implanted in culprit lesion (mm)	3.17 ± 0.44	2.99 ± 0.41*	2.92 ± 0.44*	
Stent length implanted in culprit lesion (mm)	22 ± 9	26 ± 16	22 ± 9	
TIMI grade 3 after PCI	76 (82.6)	73 (79.3)	74 (80.4)	0.849
Peak CK	3168 ± 2755	2322 ± 2035*	2585 ± 3080	

SBP: systolic blood pressure; HR: heart rate; MI: myocardial infarction; STEMI: ST-elevation myocardial infarction; PG: plasma glucose; WBC: white blood cell; TIMI: thrombolysis in myocardial infarction; PCI: percutaneous coronary intervention; CK: creatine phosphokinase. *P < 0.05 versus low K; ^†^ *P* < 0.05 versus mid K.

**Table 3:** Clinical characteristics of the patients at followup.

	Low K (<3.9)	Mid K (≥3.9, <4.3)	High K (≥4.3)	*P* value
	*n* = 92	*n* = 92	*n* = 92	
Time from PCI to followup (day)	196 ± 53	206 ± 77	187 ± 65	
Target lesion revascularization (%)	14 (15.2)	21 (22.8)	29 (31.5)	0.0321
T-C at followup (mg/dL)	175 ± 40	168 ± 29	172 ± 38	
HDL-C at followup (mg/dL)	47 ± 10	49 ± 12	47 ± 11	
LDL-C at followup (mg/dL)	102 ± 37	94 ± 25	100 ± 33	
TG at followup (mg/dL)	148 ± 104	135 ± 67	141 ± 63	
Active medical treatment				
Aspirin	92 (100)	91 (98.9)	92 (100)	0.367
ACE-I/ARB	79 (85.9)	81 (88.0)	71 (77.2)	0.108
Beta-blocker	27 (29.3)	29 (31.5)	33 (35.9)	0.629
CaCB	29 (31.5)	27 (29.3)	26 (28.3)	0.886
Nitrate	16 (17.4)	16 (17.4)	21 (22.8)	0.558
Diuretics	14 (15.2)	12 (13.0)	18 (19.6)	0.469
Statin	71 (77.2)	67 (72.8)	61 (66.3)	0.254

PCI: percutaneous coronary intervention; T-C: total cholesterol; HDL-C: high-density lipoprotein cholesterol; LDL-C: low-density lipoprotein cholesterol; TG: triglyceride; ACE-I: angiotensin converting enzyme inhibitor; ARB: angiotensin receptor blocker; CaCB: calcium channel blocker.

**Table 4:** Logistic analysis for the risk of target lesion revascularization.

	Univariate model		Multivariate model	
	OR (95% CI)	*P*	OR (95% CI)	*P*
Age	1.01 (0.989–1.04)	0.286	—	—
Male	1.81 (0.924–3.56)	0.0838	—	—
BMI	1.05 (0.959–1.15)	0.292	—	—
Current smoker	1.18 (0.669–2.08)	0.568	—	—
Hypertension	0.940 (0.532–1.66)	0.833	—	—
Diabetes mellitus	1.69 (0.948–3.00)	0.0751	—	—
T-C	0.996 (0.989–1.00)	0.258	—	—
HDL-C	0.985 (0.960–1.01)	0.249	—	—
LDL-C	0.998 (0.989–1.01)	0.624	—	—
TG	0.999 (0.996–1.00)	0.613	—	—
Hemoglobin A1c	1.11 (0.925–1.33)	0.266	—	—
Time from onset to admission	1.05 (1.01–1.09)	0.0162	1.04 (0.997–1.09)	0.0688
SBP on admission	1.01 (1.00–1.02)	0.0430	1.01 (1.00–1.02)	0.0550
HR on admission	1.01 (0.994–1.02)	0.353	—	—
Previous MI	0.817 (0.263–2.54)	0.726	—	—
Preinfarction angina	0.957 (0.532–1.72)	0.883	—	—
STEMI	0.488 (0.268–0.889)	0.0190	0.625 (0.330–1.18)	0.148
Anterior MI	1.19 (0.681–2.09)	0.538	—	—
Killip class >I on admission	0.749 (0.362–1.55)	0.436	—	—
Coronary multivessel involvement	1.51 (0.858–2.67)	0.152	—	—
PG on admission	1.00 (0.997–1.00)	0.948	—	—
WBC count on admission	1.00 (1.00–1.00)	0.843	—	—
Creatinine on admission	1.20 (0.823–1.74)	0.347	—	—
Hemoglobin on admission	1.03 (0.897–1.19)	0.642	—	—
Potassium on admission	1.75 (1.10–2.79)	0.0187	1.69 (1.04–2.74)	0.0360
Diameter of implanted stent	0.517 (0.306–15.5)	0.0493	0.763 (0.383–1.52)	0.442
Length of implanted stent	1.02 (0.997–1.04)	0.0978	—	—
TIMI grade 3 after PCI	1.04 (0.509–2.12)	0.916	—	—
Peak CK	1.00 (1.00–1.00)	0.363	—	—

OR: odds ratio; CI: confidence interval; BMI: body mass index; T-C: total cholesterol; HDL-C: high-density lipoprotein cholesterol; LDL-C: low-density lipoprotein cholesterol; TG: triglyceride; SBP: systolic blood pressure; HR: heart rate; MI: myocardial infarction; STEMI: ST-elevation myocardial infarction; PG: plasma glucose; WBC: white blood cell; TIMI: thrombolysis in myocardial infarction; PCI: percutaneous coronary intervention; CK: creatine phosphokinase.

**Table 5:** Target lesion revascularization according to potassium level.

	Target lesion revascularization			
	*n* (%)	OR	95% CI	*P*
Low K (<3.9) (*n* = 92)	14 (15.2)	1.00	—	—
Mid K (≥3.9, <4.3) (*n* = 92)	21 (22.8)	1.65	0.779–3.49	0.191
High K (≥4.3) (*n* = 92)	29 (31.5)	2.57	1.25–5.26	0.0103

OR: odds ratio; CI: confidence interval.

## References

[1] Clausen T (2010). Hormonal and pharmacological modification of plasma potassium homeostasis. *Fundamental and Clinical Pharmacology*.

[2] Karlsberg RP, Cryer PE, Roberts R (1981). Serial plasma catecholamine response early in the course of clinical acute myocardial infarction: relationship to infarct extent and mortality. *American Heart Journal*.

[3] Nordrehaug JE (1985). Malignant arrhythmia in relation to serum potassium in acute myocardial infarction. *American Journal of Cardiology*.

[4] Madias JE, Shah B, Chintalapally G, Chalavarya G, Madias NE (2000). Admission serum potassium in patients with acute myocardial infarction: its correlates and value as a determinant of in-hospital outcome. *Chest*.

[5] Foo K, Sekhri N, Deaner A (2003). Effect of diabetes on serum potassium concentrations in acute coronary syndromes. *Heart*.

[6] Karlsberg RP, Cryer PE, Roberts R (1981). Serial plasma catecholamine response early in the course of clinical acute myocardial infarction: relationship to infarct extent and mortality. *American Heart Journal*.

[7] Clausen T (1983). Adrenergic control of Na^+^-K^+^-homeostasis. *Acta Medica Scandinavica. Supplementum*.

[8] Monraats PS, Pires NMM, Agema WRP (2005). Genetic inflammatory factors predict restenosis after percutaneous coronary interventions. *Circulation*.

[9] Queen LR, Xu B, Horinouchi K, Fisher I, Ferro A (2000). *β*2-adrenoceptors activate nitric oxide synthase in human platelets. *Circulation Research*.

[10] Chen AF, Ren J, Miao C-Y (2002). Nitric oxide synthase gene therapy for cardiovascular disease. *Japanese Journal of Pharmacology*.

[11] Thygesen K, Alpert JS, White HD (2007). Joint ESC/ACCF/AHA/WHF Task Force for the Redefinition of Myocardial Infarction. Universal definition of myocardial infarction. *Circulation*.

[12] Garcia-Cantu E, Spaulding C, Corcos T (1996). Stent implantation in acute myocardial infarction. *American Journal of Cardiology*.

[13] Pasceri V, Patti G, Speciale G, Pristipino C, Richichi G, Di Sciascio G (2007). Meta-analysis of clinical trials on use of drug-eluting stents for treatment of acute myocardial infarction. *American Heart Journal*.

[14] Schampaert E, Cohen EA, Schlüter M (2004). The Canadian study of the sirolimus-eluting stent in the treatment of patients with long de novo lesions in small native coronary arteries (C-SIRIUS). *Journal of the American College of Cardiology*.

[15] Takaichi K, Takemoto F, Ubara Y, Mori Y (2007). Analysis of factors causing hyperkalemia. *Internal Medicine*.

[16] Masuo K, Lambert GW, Esler MD, Rakugi H, Ogihara T, Schlaich MP (2010). The role of sympathetic nervous activity in renal injury and end-stage renal disease. *Hypertension Research*.

[17] Jamerson KA, Julius S, Gudbrandsson T, Andersson O, Brant DO (1993). Reflex sympathetic activation induces acute insulin resistance in the human forearm. *Hypertension*.

[18] Hsiao PG, Hsieh CA, Yeh CF (2012). Early prediction of acute kidney injury in patients with acute myocardial injury. *Journal of Critical Care*.

[19] Brown MJ, Brown DC, Murphy MB (1983). Hypokalemia from beta2-receptor stimulation by circulating epinephrine. *The New England Journal of Medicine*.

[20] Solomon CG, Lee TH, Cook EF (1989). Comparison of clinical presentation of acute myocardial infarction in patients older than 65 years of age to younger patients: The Multicenter Chest Pain Study Experience. *American Journal of Cardiology*.

[21] Culić V, Eterović D, Mirić D, Silić N (2002). Symptom presentation of acute myocardial infarction: influence of sex, age, and risk factors. *American Heart Journal*.

[22] Grossman SA, Brown DFM, Chang Y (2003). Predictors of delay in presentation to the ED in patients with suspected acute coronary syndromes. *American Journal of Emergency Medicine*.

[23] Ho KK, Lee SW, Ooi SBS, Lateef F, Lim SH, Anantharaman V (2002). Acute coronary syndrome—factors causing delayed presentation at the Emergency Department. *Annals of the Academy of Medicine Singapore*.

[24] Fujimoto H, Tao S, Masuda J (2008). The efficacy of bare metal stent implantation for patients with acute myocardial infarction in the drug-eluting stent era. *Journal of Cardiology*.

[25] Shugman IM, Hee L, Mussap CJ (2013). Bare-metal stenting of large coronary arteries in ST-elevation myocardial infarction is associated with low rates of target vessel revascularization. *American Heart Journal*.

[26] Sadeghi HM, Stone GW, Grines CL (2003). Impact of renal insufficiency in patients undergoing primary angioplasty for acute myocardial infarction. *Circulation*.

[27] Okura H, Taguchi H, Kubo T (2007). Impact of arterial remodelling and plaque rupture on target and non-target lesion revascularisation after stent implantation in patients with acute coronary syndrome: an intravascular ultrasound study. *Heart*.

[28] Cristea E, Stone GW, Mehran R, Kirtane AJ, Brener SJ (2011). Changes in reference vessel diameter in ST-segment elevation myocardial infarction after primary percutaneous coronary intervention: implications for appropriate stent sizing. *American Heart Journal*.

[29] Choi S-Y, Maehara A, Cristea E (2012). Usefulness of minimum stent cross sectional area as a predictor of angiographic restenosis after primary percutaneous coronary intervention in acute myocardial infarction (from the HORIZONS-AMI Trial IVUS substudy). *American Journal of Cardiology*.

[30] Nordrehaug JE, Von Der Lippe G (1983). Hypokalaemia and ventricular fibrillation in acute myocardial infarction. *British Heart Journal*.

[31] Nordrehaug JE, Johannessen KA, Von Der Lippe G (1985). Serum potassium concentration as a risk factor of ventricular arrhythmias early in acute myocardial infarction. *Circulation*.

[32] Goyal A, Spertus JA, Gosch K (2012). Serum potassium levels and mortality in acute myocardial infarction. *The Journal of the American Medical Association*.

[33] Hoit BD, Suresh DP, Craft L, Walsh RA, Liggett SB (2000). *β*2-Adrenergic receptor polymorphisms at amino acid 16 differentially influence agonist-stimulated blood pressure and peripheral blood flow in normal individuals. *American Heart Journal*.

[34] Masuo K, Katsuya T, Fu Y, Rakugi H, Ogihara T, Tuck ML (2005). *β*2-adrenoceptor polymorphisms relate to insulin resistance and sympathetic overactivity as early markers of metabolic disease in nonobese, normotensive individuals. *American Journal of Hypertension*.

[35] Chen Y, Lipkowitz MS, Salem RM (2010). Progression of chronic kidney disease: adrenergic genetic influence on glomerular filtration rate decline in hypertensive nephrosclerosis. *American Journal of Nephrology*.

